# Deep Spread Multiplexing and Study of Training Methods for DNN-Based Encoder and Decoder

**DOI:** 10.3390/s23083848

**Published:** 2023-04-10

**Authors:** Minhoe Kim, Woongsup Lee

**Affiliations:** 1 Computer and Information Science Department, Korea University, Sejong-ro, Sejong 2511, Republic of Korea; 2 Graduate School of Information, Yonsei University, Seoul 03722, Republic of Korea

**Keywords:** deep spread multiplexing, autoencoder, deep learning, SCMA

## Abstract

We propose a deep spread multiplexing (DSM) scheme using a DNN-based encoder and decoder and we investigate training procedures for a DNN-based encoder and decoder system. Multiplexing for multiple orthogonal resources is designed with an autoencoder structure, which originates from the deep learning technique. Furthermore, we investigate training methods that can leverage the performance in terms of various aspects such as channel models, training signal-to-noise (SNR) level and noise types. The performance of these factors is evaluated by training the DNN-based encoder and decoder and verified with simulation results.

## 1. Introduction

In today’s digital age, the demand for high-speed data transmission is increasing rapidly, leading to an increased strain on the limited bandwidth available. To address this challenge, multiplexing has become an indispensable technique in modern communication systems for improving the spectral efficiency of the channel. The primary goal of multiplexing is to simultaneously transmit multiple signals over the same communication channel, thereby increasing the channel utilization. Multiplexing a multiple number of signal streams to multiple resources is a promising technology for wireless networks, which must support massive connections such as for the Internet of Things (IoT) [1]. In particular, a sparse code multiple access (SCMA) scheme was proposed as one of the non-orthogonal multiple access schemes to be used in 5G mobile communication standards [2]. SCMA investigates multiple signal streams to a limited number of resources, but it has very a weak point to use in practice, because the codebook needs to be generated according to a specific factor graph. Moreover, SCMA cannot be applied to the system which has an asymmetric factor graph between signal streams and resources.

In this paper, a novel deep learning-based deep spread multiplexing (DSM) is proposed. DSM can be considered as a generalization of the SCMA system which can outperform SCMA in terms of BER performance and applicability. Furthermore, it should be noted that DSM uses the same number of resources as the SCMA system such that the performance gain of DSM can solely be beneficial. DSM has deep neural network (DNN) blocks as the encoder and decoder, which is known as an autoencoder-based [3] communication system [4,5].

Once DNNs are sufficiently trained, each DNN-based encoder and decoder can perform just like a normal encoder and decoder. Furthermore, various training methods for systems which consist of DNN-based encoders and decoders are investigated in order to improve the training procedure.

The main contributions of this paper are summarized in three points as follows:

- We proposed DSM, which spreads multiple independent data to multiple orthogonal resources using DNN-based encoder and decoder. Since DSM is built with DNNs, it can be universally applied to a system with any number of data layers and resources.
- We used simulations to show that the DSM can outperform conventional spreading schemes in terms of BER.
- We investigate various methods to train the autoencoder better in many aspects such as noise type used for generating training datasets, overfitting problem and utilization of channel information.

The remainder of this paper is organized as follows. In Section 2, we describe the system model considered in this paper. In Section 3, a deep autoencoder architecture is proposed for the DSM system and various training methods are investigated. In Section 4, we evaluate the performance of the proposed DSM and the effects of the training aspects, which is followed by a summary of the paper in Section 5.

## 2. System Model

In the DSM system, we consider a system with multiple orthogonal resources so that multiple signal streams can be multiplexed. The orthogonal resources can be considered as time resource or frequency resource (divided as subcarriers) or any orthogonal type of resources. Since DSM is developed from the SCMA model, we compare two systems and address the differences.

In the DSM system, *J* signal stream nodes and *K* resource nodes are fully related. A signal stream is multiplexed into all the resources regarding other signal streams. Then, the received signal model can be expressed as seen below [6].

(1) $$\begin{array}{ccc} y_{k} & = & h_{k}F\left(r\right)+n_{k} \end{array}$$

**Figure 1:**
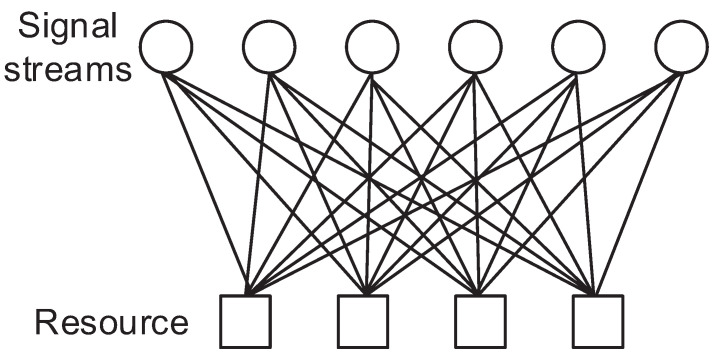
Factor graph of DSM with 6 signal streams and 4 resources.

**Figure 2:**
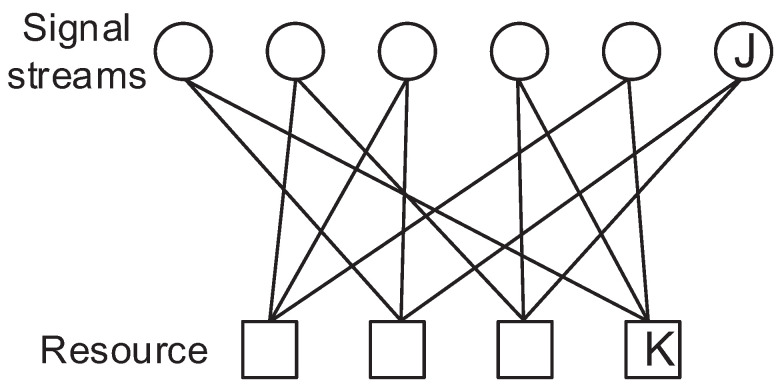
Factor graph of SCMA with 6 signal streams and 4 resources.

Here, $h_{k}$ is the channel coefficient for the resource *k* which can be considered as orthogonal subcarriers in the OFDM system or spatial layers in MIMO, r is the signal vector of $r_{j}$ which is the signal at the *j*-th stream, F(·) is the constellation mapper (encoder) regarding r and $n_{k}$ is the noise for resource *k* which follows the distribution of Gaussian with a mean of 0 and a variance of $\sigma^{2}$. The signal factor graph of SCMA and DSM is depicted in Figure 1 and Figure 2. As can be seen by comparing these figures, the signal streams are multiplexed by DNN which is different from the SCMA system, in which only some of the signal streams are summed to a resource. Therefore, all received signals are required to decode a signal stream since all the signal streams have a relation with rest of the signal streams.

We propose a deep neural network-based encoder and decoder which are inspired by the denoising autoencoder (DAE), which is the most well-known generative model in deep learning [3]. Originally, the DAE is used for denoising the corrupted data such as recovering the corrupted images into clear images; however, it has been adopted for wireless communication systems [7]. The proposed DSM is composed of a basic DNN unit formed of multiple repetitive hidden layers. Each hidden layer of a basic DNN unit is composed of a weight matrix, $W_{l}$, a bias vector, $b_{l}$ and an activation function, $\phi_{l}$, where l denotes the index of the hidden layer. In our proposed scheme, a rectified linear unit (ReLU) is used for the activation function [8]. Then, a basic DNN unit with *L* hidden layers can be represented as $\phi_{L}\left(W_{L}\phi_{L-1}\left(\cdots \phi_{1}\left(W_{1}r+b_{1}\right)\cdots \right)+b_{L}\right)$. The encoder and decoder of the DAE can be built using this DNN unit; the structure of the encoder and decoder are explained in detail in the following section.

## 3. DSM Training Methods

In this section, we present BER minimization for the DSM system which can be viewed as the generalization of the SCMA system. Furthermore, we investigate various training methods such as generation of training datasets for different types of noise, investigate overfitting by applying a dropout scheme and propose methods to utilize the channel information, i.e., other than the AWGN channel. The principle of the training procedure is similar as in [9], which proposed an autoencoder-based SCMA.

### 3.1. BER Minimization in DSM System

The role of the encoder in the DSM is to encode the input information bits into the constellation plane of the resource regarding all other information bits that are multiplexed. Furthermore, the decoder in the DSM decodes the received signal at resources and reconstructs the signal regarding all the multiplexed signals of the resources. We build the encoder with a DNN using multiple layers of perceptrons where each layer consists of an FC-layer, a batch-normalization layer and an activation layer in which we use a ReLU layer. Then, the $L_{f}$-layered DNN-based encoder of the DSM can be mathematically expressed as follows:

(2) $$f\left(r\right)=\phi_{L_{f}}\left(\right|W_{L_{f}}^{f}\phi_{L_{f}-1}(\cdots \phi_{1}\left(\right|W_{1}^{f}r+b_{1}^{f}{|}_{norm})\cdots )+b_{L_{f}}^{f}{|}_{norm})$$

**Figure 3:**
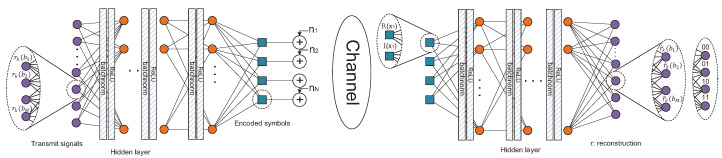
System model for DSM system.

Here, r is the original symbol, $W_{l}^{f}r+b_{l}^{f}$ is the *l*-th FC layer with weight and bias, ${\left|\cdot \right|}_{norm}$ refers to the batch norm layer and ϕ is the activation layer. Furthermore, the DNN-based decoder of the DSM system has the similar structure as the encoder. We assume that the decoder is composed of $L_{g}$ sub-blocks like the encoder such that the output of the decoder, g(y), can be expressed as

(3) $$g\left(y\right)=\phi_{L_{g}}\left(\right|W_{L_{g}}^{g}\phi_{L_{g}-1}(\cdots \phi_{1}\left(\right|W_{1}^{g}y+b_{1}^{g}{|}_{norm})\cdots )+b_{L_{g}}^{g}{|}_{norm}),$$

where y denotes the input of the decoder and $W_{l_{g}}^{g}$ and $b_{l_{g}}^{g}$ are the weights and bias for the $l_{g}$-th FC of the decoder, respectively. The detailed description of the DNN structure is depicted in Figure 3.

We follow a similar training procedure as a training procedure in the D-SCMA system. The loss function of the autoencoder structure in the DSM system can be defined as the mean squared error (MSE) between original signals and the estimation signals $\hat{r}$, which are denoted as the first input parameter and second input parameter of L(,), respectively.

(4) $$\min_{\theta_{g}}L\left(r,g\left(y;\theta_{g}\right)\right)=||r-g\left(y;\theta_{g}\right){\left|\right|}_{2}$$

(5) $$\min_{\theta_{f},\theta_{g}}L\left(r,\hat{r}\right)=L\left(r,g\left(f\left(r;\theta_{f}\right);\theta_{g}\right)\right)=||r-g\left(f\left(r;\theta_{f}\right);\theta_{g}\right){\left|\right|}_{2}$$

Equation (5) is the loss function for the end-to-end learning of the DSM system, which updates the DNN parameters of the encoder and decoder simultaneously. The parameters θ=(W,b) can be updated by the stochastic gradient descent (SGD) variant algorithm, e.g., Adam optimizer [10], which can be expressed as the following equation.

(6) $$\theta^{+}:=\theta -\alpha ▿L\left(r,\hat{r};\theta \right)$$

**Figure 4:**
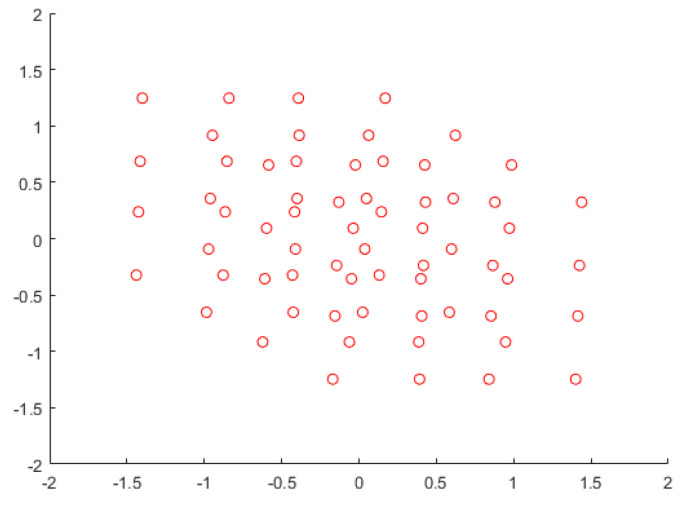
SCMA constellation points.

**Figure 5:**
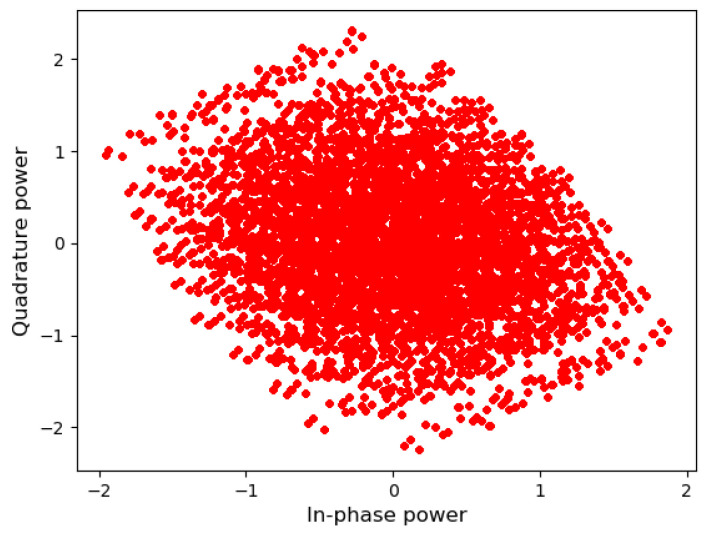
DSM constellation points.

**Figure 6:**
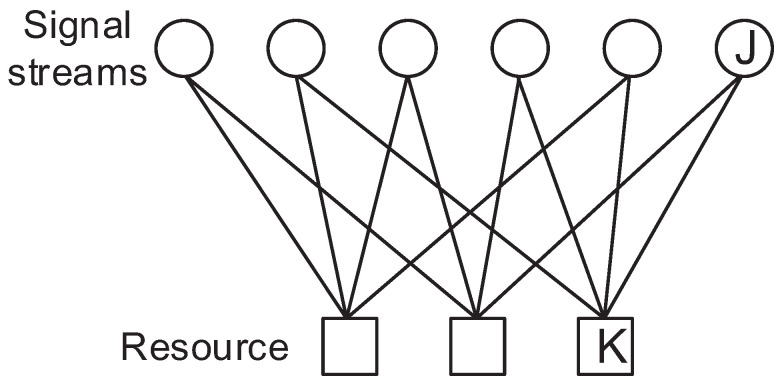
Factor graph of 6 by 3 SCMA systems.

**Figure 7:**
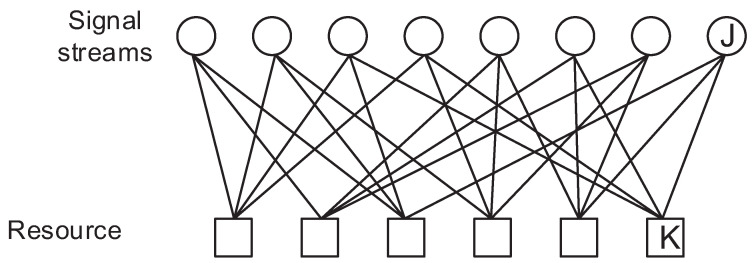
Factor graph of 8 by 6 SCMA systems.

The major difference between SCMA and DSM is that the total number of possible constellation points is $4^{3}=64$ for SCMA and $4^{6}=4096$ for DSM, as shown in Figure 4 and Figure 5, respectively. Figure 4 looks like a cloud of points because it has relatively many points in a limited space; however, it can still be decoded accurately by examining the received signal of other resources. The same framework can be applied to different configurations such as Figure 6 and Figure 7 as well.

### 3.2. Generation of Trainset with Different Noise Types

In the training phase of the DNN-based encoder and decoder, an intentionally corrupted training dataset is generated, so that the DNN encoder and decoder can operate well in the channel involved environment. As can be seen from [9], too large a corruption level, which is defined as noise power over average transmit signal power $\eta =\sigma_{train}^{2}/{E[|x|}^{2}]$ for training data, degrades the BER performance of the DNN, and too small a corruption level also degrades the BER performance since the DNN cannot learn exact boundaries in the constellation plane.

In order to generate a good quality training dataset, we propose truncated Gaussian noise as the data corruption. Truncated Gaussian distribution has the same probability distribution as the Gaussian distribution, but when the value exceeds a certain threshold, the value is regenerated according to the Gaussian distribution so that all generated data are truncated under the threshold. Truncated Gaussian noise can be expressed mathematically as seen below.

(7) $$n∼N\left(0,\sigma_{train}^{2}\right),\;\mathrm{if}\;\left|n\right|>2\sigma_{train}\;\mathrm{regenerate}$$

**Figure 8:**
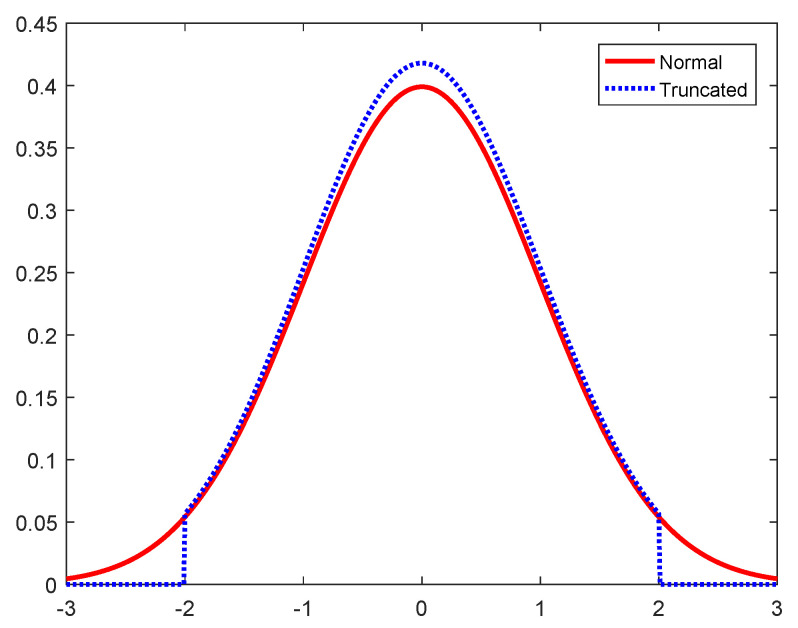
Truncated Gaussian distribution and Gaussian distribution.

The difference of truncated Gaussian distribution and Gaussian distribution is plotted in Figure 8, where $\sigma_{train}=1$. When the noise is truncated within a certain range, we can expect that the bad training samples, i.e., received signals at constellation points which overlap the boundaries of the original symbol detection region, can be decreased.

### 3.3. Overfitting

In deep learning, the training samples are usually used multiple times because a higher number of training epochs can lead to a more accurate DNN model. Such training method induces overfitting, which degrades the accuracy of the DNN when a new sample of data is evaluated. In order to overcome such defects of the overfitting problem, dropout is the most widely used training method.

The dropout scheme can be divided into two stages. First, in the training stage, some portion of the hidden nodes are considered as “dead” nodes, in other words, the connections regarding the “dead” nodes are eliminated. The “dead” nodes are selected randomly for every batch update while fixing the dropout rate. In the second stage, the dropped nodes become alive in the evaluation stage. Therefore, the new training samples are evaluated with the dropout rate 100%. Here, we investigate the effect of dropout scheme in training DSM.

### 3.4. Utilization of Channel Information

**Figure 9:**
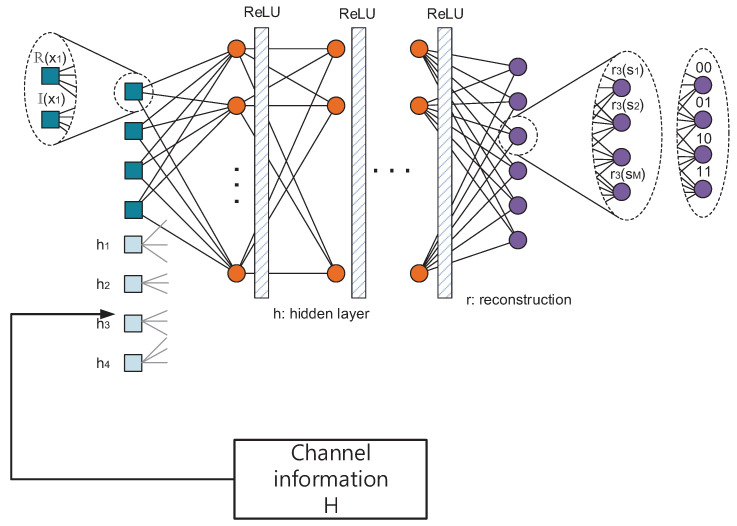
Channel information-aided DSM.

In the previous simulations, we have considered the AWGN channel, which does not require specific channel information. We propose a DNN model such that it can utilize the channel information, such as a Rayleigh multipath fading channel. When the system uses *K* orthogonal resources, the total number of channel coefficients is *K*, for instance, four channel coefficients can be utilized in six by four DSM systems.As depicted in Figure 9, the number of input nodes is increased as high as the number of channel information. Then, the DNN is able to reconstruct the signal given all the information from the input node, so we can expect that if channel information is processed properly by the DNN, it can be helpful to decode signals that experience a multipath fading channel. Furthermore, the decoder is also fed with the channel information which is concatenated with the received signal so that DNNs are able to utilize channel information to both encode and decode adaptively to the varying channel environments.

Then, the channel information-aided DSM loss function can be defined as seen below.

(8) $$\begin{array}{ccc} L(\left[r,h\right],\hat{r};\theta_{f},\theta_{g}) & = & L(\left[r,h\right],g\left(\left[y,h\right];\theta_{g}\right))\\ & = & ||\left[r,h\right]-g\left(\left[hf\left(r;\theta_{f}\right)+n,h\right];\theta_{g}\right){\left|\right|}_{2}, \end{array}$$

## 4. Performance Evaluation

In this section, we evaluate the BER performance of the DSM and compare it with the conventional SCMA and D-SCMA to verify the training methods mentioned in the previous sections. First, different types of noise are used for generating the training dataset; moreover, the channel information-aided DSM system is evaluated. For both DSM and D-SCMA simulations, we use 4-QAM modulation, and 50,000 training samples are used to train for 10 epochs with a 400 batch size, which was enough to confirm the convergence of loss function. Furthermore, 100,000 test samples were used to test for each SNR environment. The learning rate, which refers to the step size of the Adam algorithm, is set to 0.0001 while the learning rate of 0.001 shows similar performance. However, we used 0.0001 to ensure that the cost function converges. D-SCMA has six hidden layers with 32 hidden nodes per layer for encoder and five hidden layers with 512 hidden nodes per layer for decoder. DSM has five hidden layers with 512 hidden nodes per layer for both encoder and decoder. To address complex signal, we separated a complex number into two independent real numbers and combine them at the end. Throughout the simulations, the average transmit power of DSM, D-SCMA and SCMA were set to be equivalent. Nevertheless, it should be noted that the complicated structure due to the DNNs could lead to higher power consumptions for DNN-based methods. For the performance comparison, we consider the message-passing algorithm (MPA) [11,12] detection method, which is know to be near the optimum detection method and D-SCMA for the baseline.

**Figure 10:**
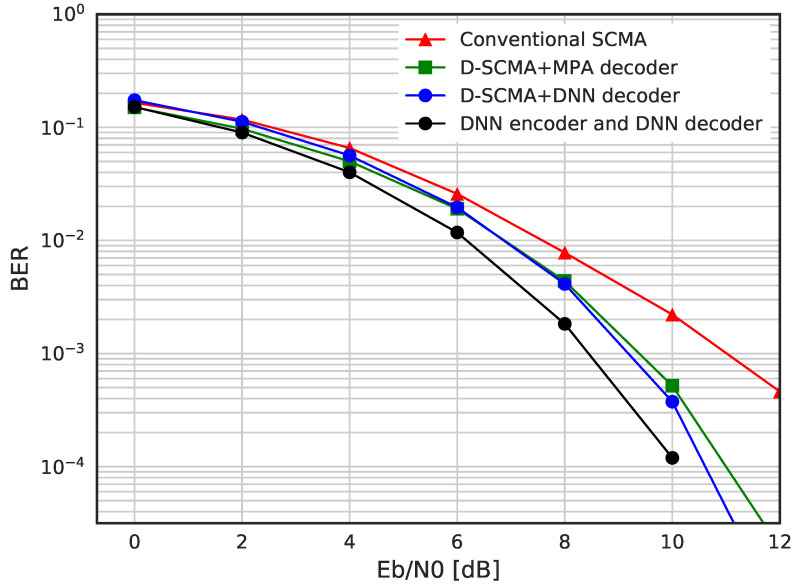
BER performance for conventional SCMA, D-SCMA and DSM.

In Figure 10, we evaluate the BER performance of conventional SCMA, DNN-based SCMA and DSM. We can see that the proposed DSM (denoted with a black circle marker) outperforms the rest of schemes based on SCMA, which implies that fully connected multiplexing is better than a sparsely connected system. DSM has shown about 1 dB SNR improvement compared with other schemes in the AWGN channel.

**Figure 11:**
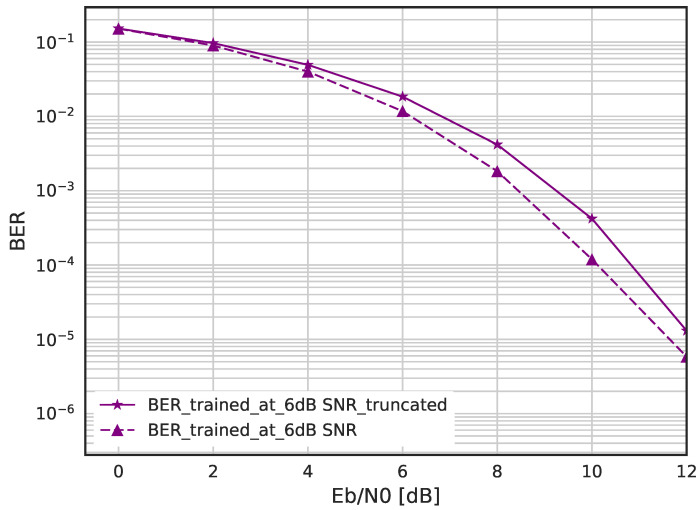
BER performance for Gaussian and truncated Gaussian noise when η = −6 dB.

**Figure 12:**
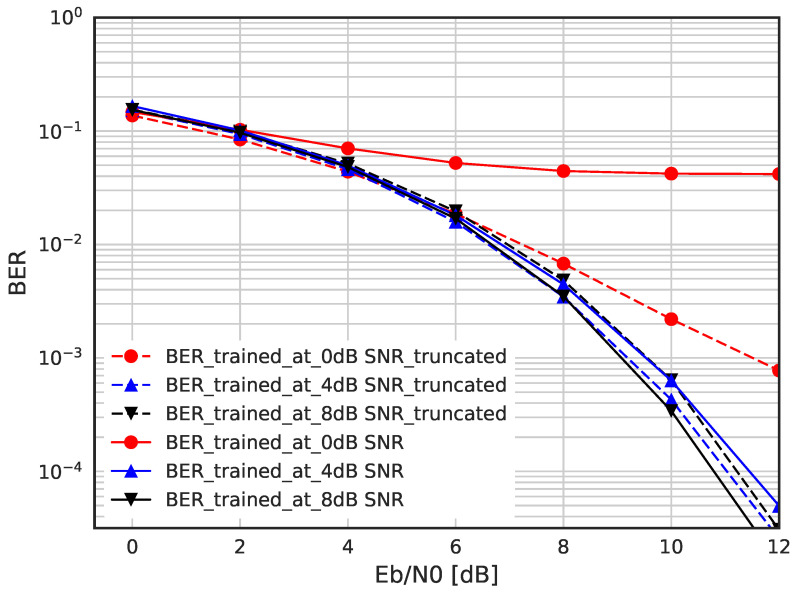
BER performance for Gaussian and truncated Gaussian noise when η = 0, −4, −8 dB.

In Figure 11 and Figure 12, we evaluate the BER performance when the DSM is trained with Gaussian noise and truncated Gaussian noise. Figure 11 shows the BER when the DSM is trained with corruption level η = −6 dB using Gaussian and truncated Gaussian noise, where each are the best-performing DNNs among each group. It should be noted that the truncated threshold is fixed as $2\sigma_{train}$ and η = −6 dB was found by exhaustive search. We can see that the DSM trained with pure Gaussian is better than that with truncated Gaussian. On the other hand, for large corruption levels such as η = 0 or −4 dB, as shown in Figure 12, the DSM with truncated Gaussian noise is trained better than that with pure Gaussian noise.

**Figure 13:**
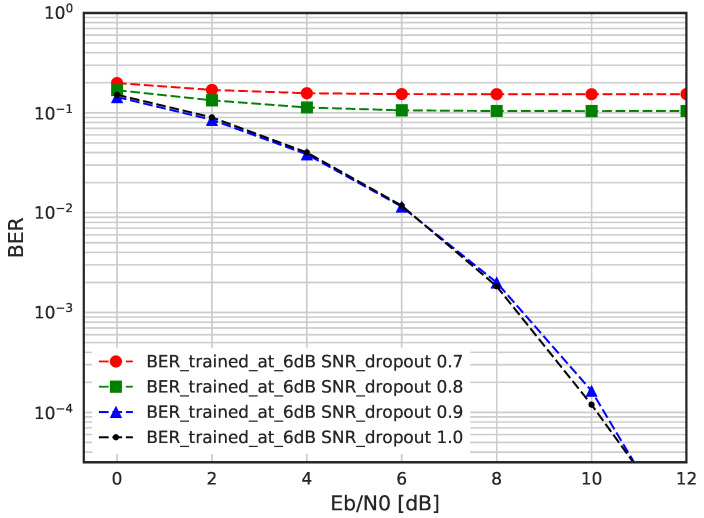
BER performance of DSM with dropout.

In Figure 13, we evaluate BER performance of DSM when dropout is applied, which prevents the overfitting. As we can see from the result, as the dropout rate decreases below 0.9, DNN cannot function as encoder and decoder. Therefore, we can see that the proposed DNN based encoder and decoder does not suffer from overfitting problem. The use of enough training data and the proper stoppage point on training epochs have helped to avoid overfitting problem.

**Figure 14:**
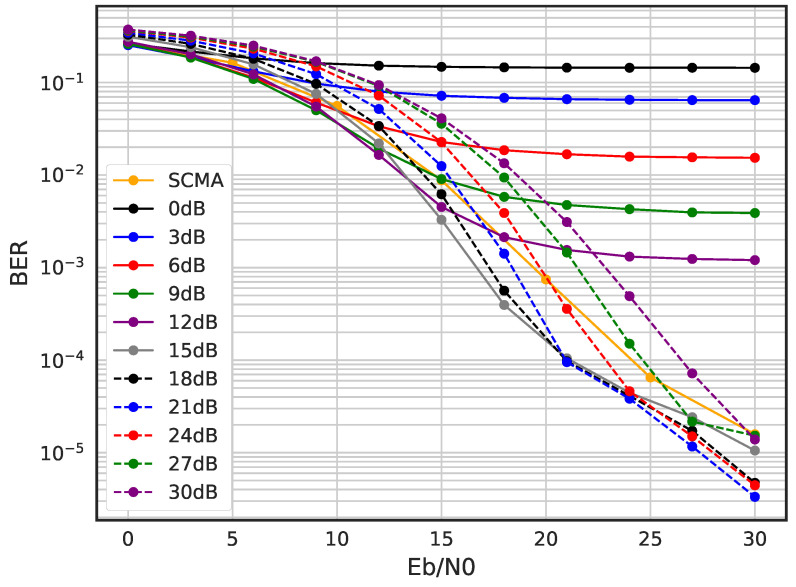
BER performance for channel information-aided DSM with different corruption levels.

In Figure 14, we evaluate BER performance of channel information-aided DSM and compare it with SCMA in the Rayleigh fading channel environment. Unlike the AWGN channel results, the BER performance of the trained DSM varies heavily depending on the corruption level. Since the multipath fading channel has a much larger variance compared to the AWGN channel, the proper selection of corruption level is very important and, as can be seen from the results, the −15 dB, −18 dB and −21 dB corruption levels are best for training the DNN-based encoder and decoder.

**Figure 15:**
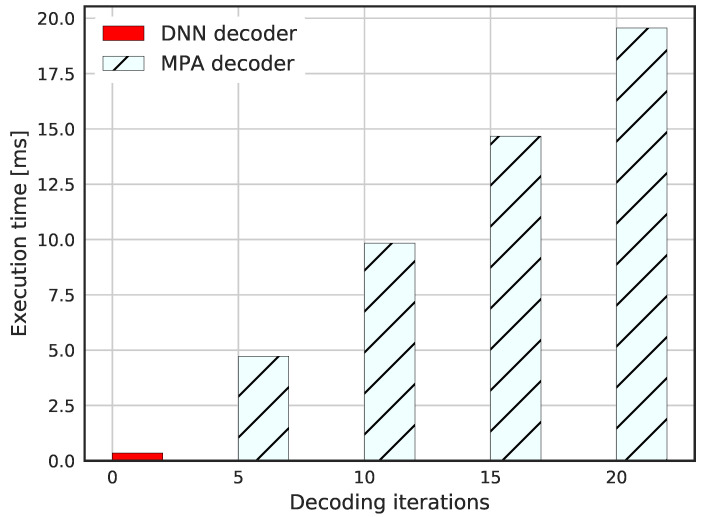
Complexity comparison for DNN-based SCMA and conventional MPA decoder.

In Figure 15, the complexity of conventional MPA detection and the DNN-based detector are evaluated. The complexity of the conventional MPA scheme is proportional to the number of message-passing update iteration, *I*, which can be expressed as $O(I\cdot \left(d_{c}^{2}\cdot M^{d_{c}}+d_{c}^{2}JM/K\right))$ [13] while the DNN decoder has $O(L\cdot \left(d_{c}K\right))$ where $d_{c}$ refers to the number of superposed layers in a resource. As can be seen, the experimental results verify the practical usage of the DNN-based encoder and decoder.

## 5. Conclusions

In this paper, we proposed a novel multiplexing scheme for the generalization of the SCMA system. An autoencoder architecture is adopted to implement the DNN-based encoder and decoder so that the MSE of original signal and estimated signal can be minimized. Furthermore, various training methods were investigated to improve the performance of the trained DSM system. Gaussian noise and truncated Gaussian noise were used for generating the training dataset and the overfitting problem was studied. Lastly, a method to use channel information for the DNN-based encoder and decoder was proposed. Through simulation results, we showed that any number of signal streams and resources can be used in the DSM system and traits of the truncated Gaussian noise-based training dataset were discovered. Moreover, the overfitting problem was revealed to be of no harm to the DNN-based encoder and decoder system. Channel utilization for the DSM was successfully performed by the proposed architecture.

## Sample Availability

Simulation codes are available from the authors upon reasonable request.

## References

[1] Ding Z., Liu Y., Choi J., Sun Q., Elkashlan M., Chih-Lin I., Poor H.V. (2017). Application of non-orthogonal multiple access in LTE and 5G networks. IEEE Commun. Mag..

[2] (2016). Sparse Code Multiple Access (SCMA) for 5G Radio Transmission.

[3] Vincent P., Larochelle H., Lajoie I., Bengio Y., Manzagol P.-A. (2010). Stacked denoising autoencoders: Learning useful representations in a deep network with a local denoising criterion. J. Mach. Learn. Res..

[4] Wu D., Nekovee M., Wang Y. (2020). Deep learning-based autoencoder for m-user wireless interference channel physical layer design. IEEE Access.

[5] O’shea T., Hoydis J. (2017). An introduction to deep learning for the physical layer. IEEE Trans. Cogn. Commun. Netw..

[6] Nikopour H., Baligh H. Sparse code multiple access. Proceedings of the 2013 IEEE 24th Annual International Symposium on Personal, Indoor, and Mobile Radio Communications (PIMRC).

[7] Zou C., Yang F., Song J., Han Z. (2021). Channel autoencoder for wireless communication: State of the art, challenges, and trends. IEEE Commun. Mag..

[8] Nair V., Hinton G.E. Rectified linear units improve restricted boltzmann machines. Proceedings of the 27th International Conference on Learning Representations (ICLR).

[9] Kim M., Kim N., Lee W., Cho D. (2018). Deep learning-aided SCMA. IEEE Commun. Lett..

[10] Kingma D.P., Ba J. Adam: A method for stochastic optimization. Proceedings of the 3rd International Conference on Learning Representations (ICLR).

[11] Kschischang F.R., Frey B.J., Loeliger H.-A. (2001). Factor graphs and the sum-product algorithm. IEEE Trans. Inf. Theory.

[12] Ameur W.B., Mary P., Dumay M., Hélard J.F., Schwoerer J. Performance study of MPA, Log-MPA and MAX-Log-MPA for an uplink SCMA scenario. Proceedings of the IEEE 26th International Conference on Telecommunications (ICT).

[13] Wei F., Chen W. A low complexity scma decoder based on list sphere decoding. Proceedings of the IEEE Global Communications Conference (GLOBECOM).

